# Editorial of the Special Issue ‘Nano-Optics and Nano-Optoelectronics: Challenges and Future Trends’

**DOI:** 10.3390/nano14020169

**Published:** 2024-01-12

**Authors:** Hai-Zhi Song

**Affiliations:** 1 Quantum Research Center, Southwest Institute of Technical Physics, Chengdu 610041, China; hzsong@uestc.edu.cn; Tel.: +86-158-2823-9155; 2 Institute of Fundamental and Frontier Sciences, University of Electronic Science and Technology of China, Chengdu 610054, China; 3 State Key Laboratory of High Power Semiconductor Lasers, Changchun University of Science and Technology, Changchun 130013, China

Through nano-optics and nano-optoelectronics, we can investigate the characteristics of light at the nanometer scale and the interaction of nanometer-scale objects with light. This interdisciplinary field combines advancements in optics/photonics and nanotechnology, making it an essential area in science and technology. In the past decade, extensive research has been conducted in this field, demonstrating promising prospects for applications such as optical communication, interconnection, storage, sensing and imaging, optics, display and lighting, medicine, security, and green energy. Most recently, through the design and modulation of the optical and electronic properties of nanomaterials and nanostructures, such as quantum dots, single- or multilayer films, metasurfaces, metalenses, etc., it has been anticipated that significant advancements could be made, allowing us to face new challenges using nanoscale photonic systems, nano-optoelectronic devices, nano-optical techniques, etc.

To explore the latest developments and challenges and the future trends in nano-optics and nano-optoelectronics, this Special Issue compiles sixteen papers dedicated to very recent advances in nanostructures and nanomaterials. In these papers, novel nanostructures and nanomaterials, such as metastructures [1,2,3], metalenses [4,5], nanoparticles [6,7], layered 2D materials [8,9], and nano-semiconductors [10,11], are presented in detail. Due to their exceptional properties, these nanostructures/nanomaterials offer a diverse range of novel applications in photodetectors [1,9,11], solar cells [2,9,10], modulators [1,3,8], light emitters [1,7], optoelectronic integration [5,6], super-resolved imaging [4,5], nanofabrication [4], etc. This Special Issue also encompasses the latest advancements in practical nano-optical and nano-optoelectronic technologies, such as femtosecond laser nano-processing [12], atomic layer coating [13], and new designs for photonic integration engineering [14]. It concludes with reviews that aim to offer comprehensive overviews of nano-related infrared absorption technology [15] and neuromorphic photonics devices [16], and to predict the futural trends in these nano-optic and nano-optoelectronic topics. In the following paragraphs, a concise summary of each article will be presented, to pique the interest of potential readers. 

One group of articles concerns the nanostructures and nanomaterials applied in optics and optoelectronics. Meta-structures are viewed as promising in the improvement of optoelectronic devices. Kanyang et al. [1] recently conducted a theoretical investigation into the integration into lithium niobate (LiNbO_3_) metasurfaces of liquid crystals (LCs), where they introduced cylinder arrays to support quasi-bound states in the continuum (quasi-BICs). The proposed structure exhibits remarkable sensitivity for tuning and other high-performance indicators, meaning that it could potentially be utilized in tunable displays, light detection and ranging (LiDAR), and spatial light modulators (SLMs). Hossain et al. [2] aimed to evaluate the performance of a wide-band octagonal perfect metamaterial absorber (PMA) for the visible wavelength spectrum. The proposed PMA exhibits remarkable absorption in both TE and TM modes and insensitivity to polarization and incident angles. Wang et al. [3] present a novel angular-dependent THz modulator that utilizes hybrid metal–graphene metastructures. Theoretical and experimental analyses show that the hybrid device offers a promising approach to applications in the field of THz technology. Metalenses serve as an advanced platform to arbitrarily control optical waves, so Shen et al. [4] employed a single-layer all-dielectric metalens based on the Pancharatnam–Berry (PB) phase to simultaneously generate and concentrate an azimuthally polarized vortex beam. It appears to have great potential for applications in super-resolution imaging, photolithography, and particle manipulation. Zhao et al. [5] propose two broadband achromatic metalenses made from a Ge_2_Sb_2_Te_5_ block on a SiO_2_ substrate. Their novel properties may provide a significant scheme for on-chip and tunable devices for NIR imaging and communication systems. Nanoparticles or quantum dots, as the 2023 Nobel Prize for Chemistry proved, have found a key role in optoelectronics and are worthy of further exploration within nanotechnology. Fernández-Martínez et al. [6] present a gain-compensated plasmonic waveguide that integrates linear chains of Ag nanoparticles on an optically active Nd^3+^-doped solid-state gain medium. Their experiments demonstrated a significant improvement in electromagnetic energy transport, and they offer new perspectives on integrated hybrid plasmonic–photonic circuits based on quantum dot platforms. Wang et al. [7] present a straightforward method for synthesizing stable heterostructures of CsPbBr_3_-quantum-dots/p-MSB-nanoplates at room temperature and atmospheric pressure; this method is synergistic in that it simultaneously tackles both the challenging issues of the unsatisfied luminous efficiency and the stability of perovskites. Two-dimensional (2D) materials are more and more attractive for nano-optoelectronic applications. To improve their terahertz applicability, Su et al. [8] propose an optically controlling broadband modulator composed of layer-dependent 2D-PtSe_2_ nanofilm. It exhibits superior surface photoconductivity in the terahertz range and has a higher plasma frequency and a lower scattering time, suggesting its suitability for terahertz modulation. Zhou et al. [9] systematically investigated the tunable properties of 2D bismuth oxyhalides (BiOX) by adjusting the number of monolayers, applying strain, and/or varying the halogen composition. The results suggest that 2D BiOX materials could be a novel candidate for nanoscale tunable photodetectors and high-performance solar cells. Semiconductors have long played important roles in nano-optics and nano-optoelectronics, and extended studies would be helpful to optimizing the function of semiconductors. Chen et al. [10] introduced Ti ions into an A_2_SnY_6_ double-perovskite structure with quantum dots (QDs). The utilization of perovskite QDs holds significant promise for developing light sources suitable for underwater optical wireless communication systems. Liu et al. [11] investigated the impact of the absorber doping concentration on the performance of an InGaAs photodiode and revealed a highly efficient method of enhancing the maximum quantum efficiency of nano-optoelectronic devices. 

Another group of articles is related to nano-optical and nano-optoelectronic technologies. Wu et al. [12] introduce a post-processing trimming method for compensating for fabrication errors on the silica cladding caused by an fs laser, while minimizing any impact on the core layer. The implementation of an fs laser to compensate for the phase bias in MZI electro-optic switches results in reduced power consumption during operation, so it is very efficient in optimizing nanophotonic processing. Astrauskytė et al. [13] discuss the feasibility of producing an anti-reflective coating on hybrid polymer micro-lenses manufactured through LDW techniques while maintaining their original geometry. Through the utilization of atomic layer deposition (ALD), this coating was able to greatly decrease reflection. It is thus suggested as an effective technique for nanomaterial fabrication. Yuan et al. [14] present a novel bandgap optimization algorithm that integrates the finite element method and topology optimization for the purpose of designing a multi-channel frequency router. Their approach exhibits generality, enabling its application across various materials and frequency ranges, and thus could serve as a novel technique for on-chip multi-channel broadband information processing.

The third group of papers are reviews concerning nano-optics and nano-optoelectronics. Surface-enhanced infrared absorption (SEIRA) spectroscopy is an innovative method that utilizes the field-amplifying properties of periodic nanostructures to enhance the vibrational signals of trace molecules. Li et al. [15] provide a comprehensive review of the SEIRA technology, covering the main trends in materials, sensitivity enhancement, bandwidth improvement, and applications. They highlight the emerging needs that have driven the development of compact and integrated infrared spectroscopy systems and chips, while also emphasizing the role of machine learning in facilitating device design and data analysis. Li et al. [16] present a comprehensive review of neuromorphic photonic devices that make use of phase-change materials (PCMs) and silicon photonic technology. They conduct a comprehensive analysis of various PCMs employed in neuromorphic devices, comparing their optical properties and discussing their applications. They emphasize that although an ideal PCM for neuromorphic photonics has not yet been identified, there are several potential solutions capable of improving the performance of these devices.

In summary, this Special Issue of *Nanomaterials* entitled “Nano-Optics and Nano-Optoelectronics: Challenges and Future Trends” compiles a series of original research articles and two review papers providing new insights into nano-optics and nano-optoelectronics and challenging the new and higher nano-optical and nano-optoelectronic technological requirements. We are confident that this Special Issue will provide the reader with a rich view of the latest prospects in the fields of nano-optics and nano-optoelectronics. 

## References

[1] Kanyang R., Fang C., Yang Q., Shao Y., Han G., Liu Y., Hao Y. (2022). Electro-Optical Modulation in High Q Metasurface Enhanced with Liquid Crystal Integration. Nanomaterials.

[2] Hossain M.J., Rahman M.H., Faruque M.R.I. (2023). An Innovative Polarisation-Insensitive Perfect Metamaterial Absorber with an Octagonal-Shaped Resonator for Energy Harvesting at Visible Spectra. Nanomaterials.

[3] Wang H., Linghu J., Wang X., Zhao Q., Shen H. (2023). Angular-Dependent THz Modulator with Hybrid Metal-Graphene Metastructures. Nanomaterials.

[4] Shen Z., Huang S. (2022). Generation of Subdiffraction Optical Needles by Simultaneously Generating and Focusing Azimuthally Polarized Vortex Beams through Pancharatnam–Berry Metalenses. Nanomaterials.

[5] Zhao L., Jiang X., Wang Z., Chen Y., Chen L., Gao B., Yu W. (2023). Broadband Achromatic Metalens for Tunable Focused Vortex Beam Generation in the Near-Infrared Range. Nanomaterials.

[6] Fernández-Martínez J., Carretero-Palacios S., Molina P., Bravo-Abad J., Ramírez M.O., Bausá L.E. (2022). Silver Nanoparticle Chains for Ultra-Long-Range Plasmonic Waveguides for Nd^3+^ Fluorescence. Nanomaterials.

[7] Wang Y., Li M.-y., Liu S., Ma Y., Sun B., Wang L., Lu H., Wen X., Liu S., Ding X. (2023). A Novel Strategy for the Synthesis of High Stability of Luminescent Zero Dimensional–Two Dimensional CsPbBr_3_ Quantum Dot/1,4-bis(4-methylstyryl)benzene Nanoplate Heterostructures at an Atmospheric Condition. Nanomaterials.

[8] Su H., Zheng Z., Yu Z., Feng S., Lan H., Wang S., Zhang M., Li L., Liang H. (2023). Optically Controlling Broadband Terahertz Modulator Based on Layer-Dependent PtSe_2_ Nanofilms. Nanomaterials.

[9] Zhou Y., Cheng B., Huang S., Huang X., Jiang R., Wang X., Zhang W., Jia B., Lu P., Song H.-Z. (2023). The Tunable Electronic and Optical Properties of Two-Dimensional Bismuth Oxyhalides. Nanomaterials.

[10] Chen W., Liu G., Dong C., Guan X., Gao S., Hao J., Chen C., Lu P. (2023). Investigation of Vacancy-Ordered Double Perovskite Halides A_2_Sn_1−x_Ti_x_Y_6_ (A = K, Rb, Cs; Y = Cl, Br, I): Promising Materials for Photovoltaic Applications. Nanomaterials.

[11] Liu H., Wang J., Guo D., Shen K., Chen B., Wu J. (2023). Design and Fabrication of High Performance InGaAs near Infrared Photodetector. Nanomaterials.

[12] Wu Y., Shang H., Zheng X., Chu T. (2023). Post-Processing Trimming of Silicon Photonic Devices Using Femtosecond Laser. Nanomaterials.

[13] Astrauskytė D., Galvanauskas K., Gailevičius D., Drazdys M., Malinauskas M., Grineviciute L. (2023). Anti-Reflective Coatings Produced via Atomic Layer Deposition for Hybrid Polymer 3D Micro-Optics. Nanomaterials.

[14] Yuan H., Zhang N., Zhang H., Lu C. (2023). A Multi-Channel Frequency Router Based on an Optimization Algorithm and Dispersion Engineering. Nanomaterials.

[15] Li D., Xu C., Xie J., Lee C. (2023). Research Progress in Surface-Enhanced Infrared Absorption Spectroscopy: From Performance Optimization, Sensing Applications, to System Integration. Nanomaterials.

[16] Li T., Li Y., Wang Y., Liu Y., Liu Y., Wang Z., Miao R., Han D., Hui Z., Li W. (2023). Neuromorphic Photonics Based on Phase Change Materials. Nanomaterials.

